# Abnormal levels of mitochondrial proteins in plasma neuronal extracellular vesicles in major depressive disorder

**DOI:** 10.1038/s41380-021-01268-x

**Published:** 2021-09-01

**Authors:** Edward J. Goetzl, Owen M. Wolkowitz, Vinod H. Srihari, Victor I. Reus, Laura Goetzl, Dimitrios Kapogiannis, George R. Heninger, Synthia H. Mellon

**Affiliations:** 1 Professor Emeritus, University of California School of Medicine, San Francisco, CA USA; 2 Weill Institute for Neurosciences and Department of Psychiatry, University of California School of Medicine, San Francisco, CA USA; 3 Department of Psychiatry, Yale University School of Medicine, New Haven, CT USA; 4 Department of Obstetrics, Gynecology and Reproductive Sciences, University of Texas Health Science Center, Houston, TX USA; 5 Intramural Research Program, National Institute on Aging, Baltimore, MD USA; 6 Department of Obstetrics, Gynecology and Reproductive Sciences, University of California School of Medicine, San Francisco, CA USA

**Keywords:** Predictive markers, Biological techniques

## Abstract

To characterize neuronal mitochondrial abnormalities in major depressive disorder (MDD), functional mitochondrial proteins (MPs) extracted from enriched plasma neuron-derived extracellular vesicles (NDEVs) of MDD participants (*n* = 20) were quantified before and after eight weeks of treatment with a selective serotonin reuptake inhibitor (SSRI). Pretreatment baseline NDEV levels of the transcriptional type 2 nuclear respiratory factor (NRF2) which controls mitochondrial biogenesis and many anti-oxidant gene responses, regulators of diverse neuronal mitochondrial functions cyclophilin D (CYPD) and mitofusin-2 (MFN2), leucine zipper EF-hand containing transmembrane 1 protein (LETM1) component of a calcium channel/calcium channel enhancer, mitochondrial tethering proteins syntaphilin (SNPH) and myosin VI (MY06), inner membrane electron transport complexes I (subunit 6) and III (subunit 10), the penultimate enzyme of nicotinamide adenine dinucleotide (NAD) generation nicotinamide mononucleotide adenylytransferase 2 (NMNAT2), and neuronal mitochondrial metabolic regulatory and protective factors humanin and mitochondrial open-reading frame of the 12S rRNA-c (MOTS-c) all were significantly lower than those of NDEVs from matched controls (*n* = 10), whereas those of pro-neurodegenerative NADase Sterile Alpha and TIR motif-containing protein 1 (SARM1) were higher. The baseline NDEV levels of transcription factor A mitochondrial (TFAM) and the transcriptional master-regulator of mitochondrial biogenesis PPAR γ coactivator-1α (PGC-1α) showed no differences between MDD participants and controls. Several of these potential biomarker proteins showed substantially different changes in untreated MDD than those we reported in untreated first-episode psychosis. NDEV levels of MPs of all functional classes, except complex I-6, NRF2 and PGC-1α were normalized in MDD participants who responded to SSRI therapy (*n* = 10) but not in those who failed to respond (*n* = 10) by psychiatric evaluation. If larger studies validate NDEV MP abnormalities, they may become useful biomarkers and identify new drug targets.

## Introduction

Major depressive disorder (MDD) is ranked by the World Health Organization as the single largest contributor to global disability [1, 2]. Despite its high prevalence and morbidity, however, diagnosing MDD is very unreliable. Further, current first-line pharmacological treatments for MDD are inadequate, as only 27% of patients remit after an initial trial and only 67% after four full trials [3]. Evaluation of new therapeutic approaches should include specific biomarkers that objectively identify the involvement of pathogenic mechanisms underlying MDD and may reveal relevant molecular targets [4, 5].

Investigations of blood-based biomarkers in MDD have begun to increase our understanding of relevant pathophysiology and alterations in the clinical course. Blood concentrations of some known neurotransmitters, tryptophan metabolites, endocrine hormones, immune cytokines, and growth and neurotrophic factors differ significantly in depression from those in matched controls [6–10]. With some exceptions, blood levels of growth and neurotrophic factors are decreased, whereas those of immune cytokines and cortisol show increases that positively correlate with poor responses to psychological and pharmacological therapy [6, 8]. Despite quantitatively striking changes in concentrations of some blood-based biomarkers in MDD, however, these alterations often have lacked disease specificity and any predictive capability regarding changes in severity or responses to treatment of the MDD. Interpretation of results of such studies sometimes was confounded by effects of concurrently administered medications. Little has been learned about new therapeutic targets. One recently emerging technology relies on analyses of blood mRNAs to track and predict the course and severity of depression and mania, as well as to identify potentially beneficial drug treatments [11].

Observations of strikingly altered number, morphology and electron-transport activity of neuronal mitochondria in major psychiatric diseases, accompanied by increases in polymorphisms, deletions and mutations of mitochondrial DNA, suggested that mitochondrial abnormalities may represent fundamental pathogenic mechanisms in MDD [12]. Such abnormalities also have been observed as changes in mitochondrial DNA copy numbers and altered mitochondrial respiratory chain enzymatic activities in stress-induced emotional disorders [13]. We have developed a platform for investigations of neuronal mitochondrial proteins (MPs) found in plasma neuron-derived extracellular vesicles (NDEVs) enriched for exosomes at levels that reflect those in brain neurons [14, 15]. Recent analyses of NDEV proteins in subjects with the first episode of psychosis (FP) revealed abnormalities in levels of mitochondrial electron transport complexes, structural components, tethering elements and neuroprotective factors relative to those of matched controls [16, 17]. Now we present here the results of studies of a similar range of MPs in NDEVs of MDD participants before and after therapy with a selective serotonin reuptake inhibitor in comparison with those of psychiatrically normal age- and sex-matched controls.

## Methods

### Baseline recruitment and study procedures

Major depressive disorder (MDD) outpatients not taking medications and mentally healthy controls were recruited by notices in flyers, bulletin boards, Craigslist, and newspapers and by clinical referrals of MDD subjects. Mean ages at initial diagnosis, sex distribution and mean days of depression in the study episode of responder and non-responder MDD patients were not significantly different (Table 1). Mean study age of mentally healthy controls matched those of MDD patients (Table 1). Diagnoses were made according to the Structured Clinical Interview for DSM IV-TR Axis I Disorders (SCID), which was the DSM version used during this study, and were confirmed by a clinical interview with a board-certified psychiatrist as described [18]. Depression symptom severity was assessed in MDD participants using the 17-item and 25-item Hamilton Depression Rating Scales (HDRS), where four MDD participants had a score lower than 17 for the first scale (two were 14, one was 13, and one was 16) and six MDD participants had a score lower than 25 for the second scale (three were 22 and three were 24), respectively, as described (Table 1) [18].

**Table 1:** Demographics and clinical characteristics of study participants.

	MDD, responder (*n* = 10)		MDD, non-responder (*n* = 10)		Healthy control (*n* = 10)
Age at diagnosis, years (mean ± SD)	25.3 ± 15.3		18.4 ± 7.0		N/A
Study age, years (mean ± SD)	39.0 ± 9.4		41.3 ± 11.6		37.5 ± 10.5
Sex, female/male	6/4		6/4		5/5
Days of current depressive episode (mean ± SD)	3179 ± 3089		5957 ± 4314		N/A
Depression rating scale	HDRS 17	HDRS 25	HDRS 17	HDRS 25	N/A
HDRS at baseline (mean ± SD)	18.0 ± 3.5	26.3 ± 3.3	19.9 ± 2.9	29.8 ± 5.3	ND
HDRS after treatment for 8 weeks (mean ± SD)	5.7 ± 4.0	7.8 ± 4.9	16.9 ± 3.4	23.6 ± 6.3	ND

HDRS is the Hamilton Depression Rating Scale; 17-variable version is the left-hand set and 25-variable version is the right-hand set. There are no significant differences between study groups for age, sex or baseline pre-treatment HDRS. Post-treatment HDRS values after eight weeks of treatment are significantly lower for responders than non-responders. *ND* Not done, *N/A* Not applicable

There were no meaningful differences among any other descriptors obtained for NR-Bsl, R-Bsl and Control groups, respectively, including race (total *n*/group = 10; four to six Caucasian, 0 to one African-American, one to two Asian, and two to three mixed race); education (years, mean ± SD): 16.9 ± 2.4 for NR-Bsl, 16.4 ± 2.8 for R-Bsl and 16.6 ± 2.2 for Control; ever smoked (%): 30 for NR-Bsl, 70 for R-Bsl and 20 for Control; and BMI (mean ± SD): 24.9 ± 4.6 for NR-Bsl, 24.5 ± 5.1 for R-Bsl and 22.8 ± 2.2 for Control.

Exclusion criteria for MDD subjects were: bipolar disorder, psychotic symptoms during their current major depressive episode or other mood disorders, any eating disorder or post-traumatic stress disorder during the month before entering the study, and substance abuse or dependence including alcohol within six months before entering the study. Co-morbid anxiety disorders (except PTSD) were not exclusionary if MDD was considered the primary diagnosis. Control participants had no history of any DSM-IV-TR Axis I disorder as confirmed by SCID interview. Further, none of the study participants had acute illnesses or infections, chronic inflammatory disorders, neurological disorders, or any other major medical conditions considered to be potentially confounding, as determined by history, physical examinations and routine blood screening. All participants were free of any psychotropic medications, including antidepressants, hormone supplements (except if needed for hypothyroidism), steroid-containing birth control or other potentially interfering medications, and had not had any vaccinations for at least six weeks prior to enrollment in the study. None was taking vitamin supplements above the U.S. recommended minimum daily allowances. Short-acting sedative-hypnotics were not allowed within one week prior to participation. On the day of each study visit, all participants had to pass a urine toxicology screen (marijuana, cocaine, amphetamines, phencyclidine, opiates, methamphetamine, tricyclic antidepressants, and barbiturates) and a urine test for pregnancy in women of child-bearing potential.

### Selective serotonin reuptake inhibitor (SSRI)-treatment

MDD participants underwent eight weeks of protocol-based open-label outpatient treatment with one of four SSRI antidepressants alone (NCT00285935, https://www.clinicaltrials.gov/). Responders received sertraline (4), escitalopram (4) or fluoxetine (2) and non-responders received sertaline (4), escitalopram (2), fluoxetine (3) or citalopram (1) at the same sertraline equivalency doses [19]. Compliance and clinical evaluations of drug tolerability were performed by a telephone check-in at the end of week 1 and an in-person check-in at the end of week 4 and week 8, at which times pill counts and plasma SSRI concentrations also were performed. Plasma SSRI concentrations were in the expected clinical range for each subject, suggesting excellent compliance. The severity of depressive symptoms, assessed by means of the HDRS, was the primary outcome measure repeated at the end of treatment (week 8). MDD participants were classified as “Responders” if their depression rating decreased by >50% from pre-treatment baseline and as “Non-Responders” if they showed lesser degrees of improvement. Ten MDD Responders, ten MDD Non-Responders and ten mentally healthy controls were selected for this investigation from a larger total study group based solely on concurrence with age range and sex (Table 1).

### Blood sampling

Participants were admitted as outpatients to the UCSF Clinical and Translational Science Institute (CTSI) between the hours of 0800 and 1100 after an overnight fast and instructed to sit quietly for 25–45 min before venipuncture for the baseline and eight-week post-treatment testing. Blood was collected into a lavender EDTA vacutainer tube, that was centrifuged at 1500 × g and 4° C for 10 min before plasma was removed, aliquoted into plastic Eppendorf tubes and stored at −80° C.

### Enrichment of plasma neuron-derived extracellular vesicles (NDEVs)

Aliquots of 0.25 mL plasma were incubated with 0.1 mL of thromboplastin D (ThermoFisher Scientific, Waltham, MA) for 30 min at room temperature, followed by addition of 0.15 mL of calcium- and magnesium-free Dulbecco’s balanced salt solution (DBS) with protease inhibitor cocktail (Roche, Indianapolis, IN) and phosphatase inhibitor cocktail (Thermo Fisher Scientific; DBS ^++^) as described [20, 21]. After centrifugation at 3000 × g for 30 minutes at 4 °C, total extracellular vesicles (EVs) were harvested from resultant supernatants by precipitation with 126 μL per tube of ExoQuick (System Biosciences, Mountain View, CA) and centrifugation at 1500 × g for 30 minutes at 4 °C.

To enrich neuron-derived EVs (NDEVs) including exosomes, replicate preparations of total extracellular vesicles were resuspended in 0.35 mL of DBS ^++^with 2.0 μg of mouse anti-human CD171 (L1CAM neural adhesion protein) biotinylated antibody (clone 5G3; eBiosciences, San Diego, CA) in 50 μL of 3% bovine serum albumin (BSA; 1:3.33 dilution of Blocker BSA 10% solution in DBS; ThermoFisher Scientific) per tube. After mixing for 60 minutes at room temperature, 10 μL of streptavidin agarose Ultralink resin (ThermoFisher Scientific) in 40 μL of 3% BSA were added to each tube followed by incubation for 30 minutes at room temperature with mixing. After centrifugation at 800 × g for 10 minutes at 4 °C and transfer to clean tubes of the supernatants containing exosomes from all sources except neurons, each pellet with NDEVs was suspended in 100 μL of cold 0.05 M glycine-HCl (pH 3.0) by gentle mixing for 30 seconds and centrifuged at 4000 × g for 10 minutes, all at 4 °C. Glycine-HCl supernatants then were transferred to clean tubes containing 25 μL of 10% BSA and 10 μL of 1 M Tris-HCl (pH 8.0) and mixed gently. An aliquot of 5 μL was removed from each tube for EV counts before addition of 370 μL of mammalian protein extraction reagent (M-PER, ThermoFisher Scientific). Resultant 0.5 mL lysates of NDEVs were frozen and thawed twice, and then stored at −80 °C. The total population of NDEV-depleted EV suspensions in initial supernatants from immunoprecipitation were re-precipitated with ExoQuick and suspended in 0.5 ml each of M-PER followed by freeze-thawing and storage at −80 °C before performing ELISAs.

To confirm the efficiency of immuno-enrichment, a portion of total initial EVs and of NDEV-depleted EV suspensions after absorption with anti-L1CAM and re-precipitation by ExoQuick for the ten control subjects also were extracted by M-PER for ELISA quantification of the exosome marker CD81 and the neuronal marker SNAP25 (AVIVA Systems Biology Corp., San Diego, CA). CD81 in the post-absorption residual EVs was a mean of 92.4% of that in the total EVs and SNAP25 in the post-absorption residual EVs was a mean of 9.1% of that in the total EVs, suggesting an approximately 10-fold enrichment of NDEVs.

To examine the possibility of MDD-related mitochondrial protein abnormalities in cells other than neurons, four mitochondrial proteins that showed major differences with MDD in extracts of NDEVs were also quantified in M-PER extracts of NDEV-depleted total EV suspensions for the R-Bsl, R-Tr and control sets (Table 2). There were no statistically significant differences between the levels of any of the mitochondrial proteins for these subsets of participants.

**Table 2:** Levels of mitochondrial proteins in total plasma exosome population after immuno-absorptive removal of NDEs.

Subject group	Mitochondrial protein, pg/ml			
	Complex I-6	SARM1	MOTS-c	NRF2
R-Tr	762 ± 84.4	2094 ± 373	405,958 ± 31,548	5288 ± 375
R-Bsl	806 ± 100	1873 ± 329	420,386 ± 30,552	5271 ± 412
Ctl	880 ± 186	1874 ± 313	428,711 ± 30,215	5375 ± 428

Each value is the mean ± SEM (*n* = 10). There are no statistically significant differences between the levels of any mitochondrial protein for these subsets of subjects.

For counting and sizing of extracellular vesicles, each suspension was diluted 1:50 in PBS. The mean diameter (nanometers) and concentration (particles per milliliter) of EVs in each suspension were determined by nanoparticle tracking analysis (NTA) using the Nanosight NS500 system with a G532nm laser module and NTA 3.1 nanoparticle tracking software (Malvern Instruments, Malvern, United Kingdom) as described [14]. Mean ± S.E.M. counts of NDEVs as in previous studies were 130 ± 4.16 × 10^9^/ml of plasma for Ctls. Comparing counts with corresponding levels of the exosome marker protein CD81 in extracts yielded Pearson Correlation Coefficients of ≥0.78 for all five sets of subjects.

### Quantification of NDEV proteins

NDEV proteins were quantified by enzyme-linked immunosorbent assay (ELISA) kits for human tetraspanning exosome marker CD81, subunit 6 of NADH-ubiquinone oxidoreductase (complex I), type 2 nuclear respiratory factor (NRF2), 16S rRNA-encoded humanin (CUSABIO by American Research Products, Waltham, MA), myosin VI (MY06), subunit 10 of cytochrome b-c1 oxidase (complex III) (Abbkine Scientific Co., Ltd. by American Research Products), PPAR γ coactivator-1α (PGC-1α), mitochondrial open-reading frame of the 12 S rRNA-c (MOTS-c) (Cloud-Clone Corp. by American Research Products), mitofusin 2 (MFN2), nicotinamide mononucleotide adenylyl transferase 2 (NMNAT2), cyclophilin D (CYPD) (MyBioSource, San Diego, CA), syntaphilin (SNPH), leucine zipper EF-hand containing transmembrane 1 protein (LETM1), Sterile Alpha and TIR motif-containing protein 1 (SARM-1) (Wuhan FineTest Biotech Co. by American Research Products) and transcription factor A mitochondrial (TFAM) (Aviva Systems Biology, San Diego, CA). The mean value for all determinations of CD81 in each assay group was set at 1.00, and relative values of CD81 for each sample were used to normalize their recovery.

The same procedures were used for first episode of psychosis (FP) and MDD patients as well as controls to isolate NDEVs and quantify their mitochondrial proteins. All ELISAs were performed by two of the investigators (EJG and LG) without knowledge of the identity of any subject.

### Statistics

After establishing that data were normally distributed, the significance of differences between baseline MDD levels and control values were calculated by an unpaired *t* test and of differences between levels after treatment and corresponding baseline values before treatment by a paired *t* test (Graphpad Holdings, LLC, San Diego, CA). Pearson Correlation Coefficient analyses were performed to assess relationships between HDRS values and NDEV mitochondrial protein levels at baseline and after treatment (Graphpad Prism 9). There were no adjustments for potential confounding variables.

## Results

Fourteen mammalian neuron mitochondrial proteins were quantified in plasma neuron-derived extracellular vesicles (NDEVs, that include exosomes) of 20 MDD participants before and after an eight-week course of SSRI therapy and in NDEVs of 10 matched controls (Table 1). Plasma NDEV levels of most of this same group of mitochondrial proteins in patients with a first episode of psychosis (FP) had been shown to differ significantly from those of age- and sex-matched mentally healthy controls [16, 17]. NDEV levels of those mitochondrial proteins that were not previously quantified for FP subjects now are presented in the figure legends for comparison with MDD data.

**Fig. 1:**
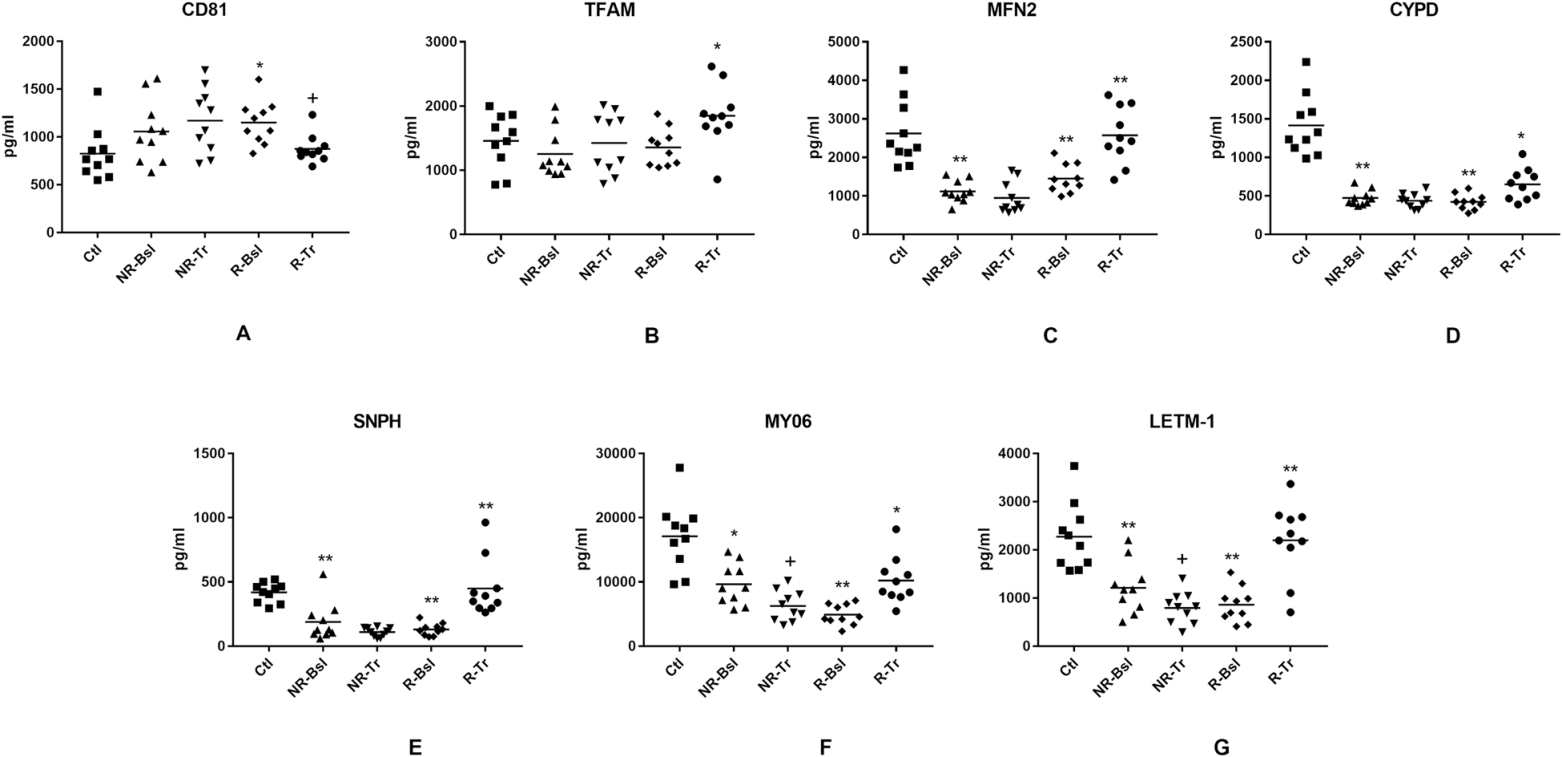
NDEV levels of proteins involved in mitochondrial dynamics and other maintenance functions. Each point represents the value for one study participant after CD81 normalization. Statistical significance of non-responder baseline (NR-Bsl) and responder baseline (R-Bsl) abnormalities were calculated relative to control values by an unpaired t test and of changes after treatment relative to respective baseline values before treatment (NR-Tr vs. NR-Bsl and R-Tr vs. R-Bsl) by a paired *t* test; for both comparisons NS not significant; +, *p* < 0.05; *, *p* < 0.01; **, *p* < 0.001. The mean ± S.E.M. (*p* value) of control subjects (Ctl), baseline of nonresponsive participants (NR-Bsl), nonresponsive participants after treatment (NR-Tr), baseline of responsive participants (R-Bsl) and responsive participants after treatment (R-Tr), respectively, were 825 ± 85 pg/ml, 1058 ± 105 pg/ml (NS), 1171 ± 107 pg/ml (NS), 1151 ± 72 pg/ml (*p* = 0.0091) and 876 ± 46 pg/ml (*p* = 0.0157) for CD81 (**A**); 1458 ± 135 pg/ml, 1255 ± 117 pg/ml (NS), 1426 ± 149 pg/ml (NS), 1357 ± 92 pg/ml (NS) and 1848 ± 152 pg/ml (0.0039) for TFAM (**B**); 2623 ± 266 pg/ml, 1120 ± 89 pg/ml (<0.0001), 950 ± 129 pg/ml (NS), 1452 ± 117 pg/ml (0.0008) and 2572 ± 234 pg/ml (0.0002) for MFN2 (**C**); 1415 ± 125 pg/ml, 473 ± 31 pg/ml (<0.0001), 438 ± 30 pg/ml (NS), 424 ± 32 pg/ml (<0.0001) and 651 ± 65 pg/ml (0.0019) for CYPD (**D**); 1676 ± 97 pg/ml, 758 ± 188 pg/ml (0.0004), 440 ± 44 pg/ml (NS), 523 ± 60 pg/ml (<0.0001) and 1797 ± 283 pg/ml (0.0008) for SNPH (**E**); 17,105 ± 1684 pg/ml, 9659 ± 1008 pg/ml (0.0013), 6269 ± 745 pg/ml (0.0473), 4915 ± 504 pg/ml (<0.0001) and 10,248 ± 1144 pg/ml (0.0017) for MY06 (**F**); and 2275 ± 221 pg/ml, 1212 ± 169 pg/ml (0.0012), 796 ± 103 pg/ml (0.0412), 860 ± 114 pg/ml (<0.0001) and 2197 ± 248 pg/ml (0.0004) for LETM-1 (**G**). Mean ± S.E.M. of levels of the same mitochondrial proteins in NDEV of previously reported sets (*n* = 10) of controls and participants with a first episode of psychosis [16], respectively, were 632 ± 18.2 pg/ml and 628 ± 16.6 pg/ml (NS) for TFAM; 1953 ± 395 pg/ml and 347 ± 138 pg/ml (0.0012) for MFN2; 1456 ± 133 pg/ml and 227 ± 33.3 pg/ml (<0.0001) for CYPD; 2188 ± 259 pg/ml and 4008 ± 371 pg/ml (0.0007) for SNPH; 9599 ± 1098 pg/ml and 11240 ± 1758 pg/ml (NS) for MY06; and 2322 ± 251 pg/ml and 485 ± 52.4 pg/ml (<0.0001) for LETM-1.

NDEV levels of neuronal proteins included the exosome marker CD81, which is used to normalize levels of all other NDEV proteins (Fig. 1). For CD81, the only alteration was a slight increase above normal controls (Ctl) of the baseline pretreatment level for those who responded to an SSRI (R-Bsl) and a return to control level after treatment (R-Tr) (Fig. 1A). The first class of NDEV mitochondrial proteins evaluated are involved in mitochondrial dynamics and functional maintenance. This class includes the prominent transcription factor TFAM, CYPD regulator of membrane potential, metabolism and pore permeability, MFN2 membrane GTPase required for mitochondrial fusion and distribution, and the tethering proteins SNPH and MY06 that anchor mitochondria to microtubules in axons and to microfilaments in pre-synaptic areas [22–26]. The only abnormality in TFAM was a slight increase for the R-Tr group above their normal R-Bsl level (Fig. 1B). In contrast, levels of both MFN2 and CYPD for the NR-Bsl and R-Bsl groups were strikingly lower than those of the Ctl groups (Fig. 1C and D). After treatment, the R-Tr level of CYPD was statistically significantly higher than that of R-Bsl and the R-Tr level of MFN2 returned completely to the Ctl level. Neither the CYPD nor MFN2 levels of the NR-Tr group were altered significantly. The pattern for NDEV group levels of the calcium channel/calcium-channel enhancing protein LETM1 resembled that of MFN2 (Fig. 1G). The levels of NR-Bsl and R-Bsl for both tethering proteins SNPH and MY06 were statistically significantly lower than those of the Ctl groups (Fig. 1E and F). The R-Tr levels, but not the NR-Tr levels, of SNPH and MY06 were statistically significantly higher than their Bsl levels. As for MDD subjects, the NDEV levels of four of the class one proteins in FP subjects were statistically significantly lower than those of controls (Fig. 1 legend and Table 3). However, differences between FP subjects and controls in NDEV levels of the tethering proteins SNPH and MY06 had patterns distinct from those of MDD subjects.

**Table 3:** Comparison of alterations in NDEV levels of mitochondrial proteins in untreated first episode of psychosis (FP) and in untreated baseline for major depressive disorder (MDD).

Class of mitochondrial analytes	FP	MDD NR-Bsl/R-Bsl
1. Dynamics and maintenance functions		
TFAM	ND	ND/ND
MFN2	↓82	↓57/45
CYPD	↓ 84	↓67/70
SNPH	↑ 183	↓55/69
MY06	ND	↓ 44/71
LETM1	↓ 79	↓47/62
2. Energy generation		
Complex I-6	↓66	↓53/80
Complex III-10	↓55	↓68/77
NMNAT 2	↓ 34	↓65/69
SARM 1	ND	↑ 443/409
3. Metabolic regulation and cellular survival		
Humanin	↓88	ND/ ↓62
MOTS-c	↓88	↓84/83
4. Mitochondrial biogenesis		
PGC1α	ND	ND/ND
NRF2	↓ 63	↓45/64

Each value represents the mean percentage change from controls for FP patients and for baseline levels of MDD groups non-responsive (NR-Bsl) and responsive (R-Bsl) to therapy. ND is no significant difference between mentally ill participants and controls. Up arrows and down arrows indicate increases and decreases of mitochondrial protein levels in mental illness, respectively.

**Fig. 2:**
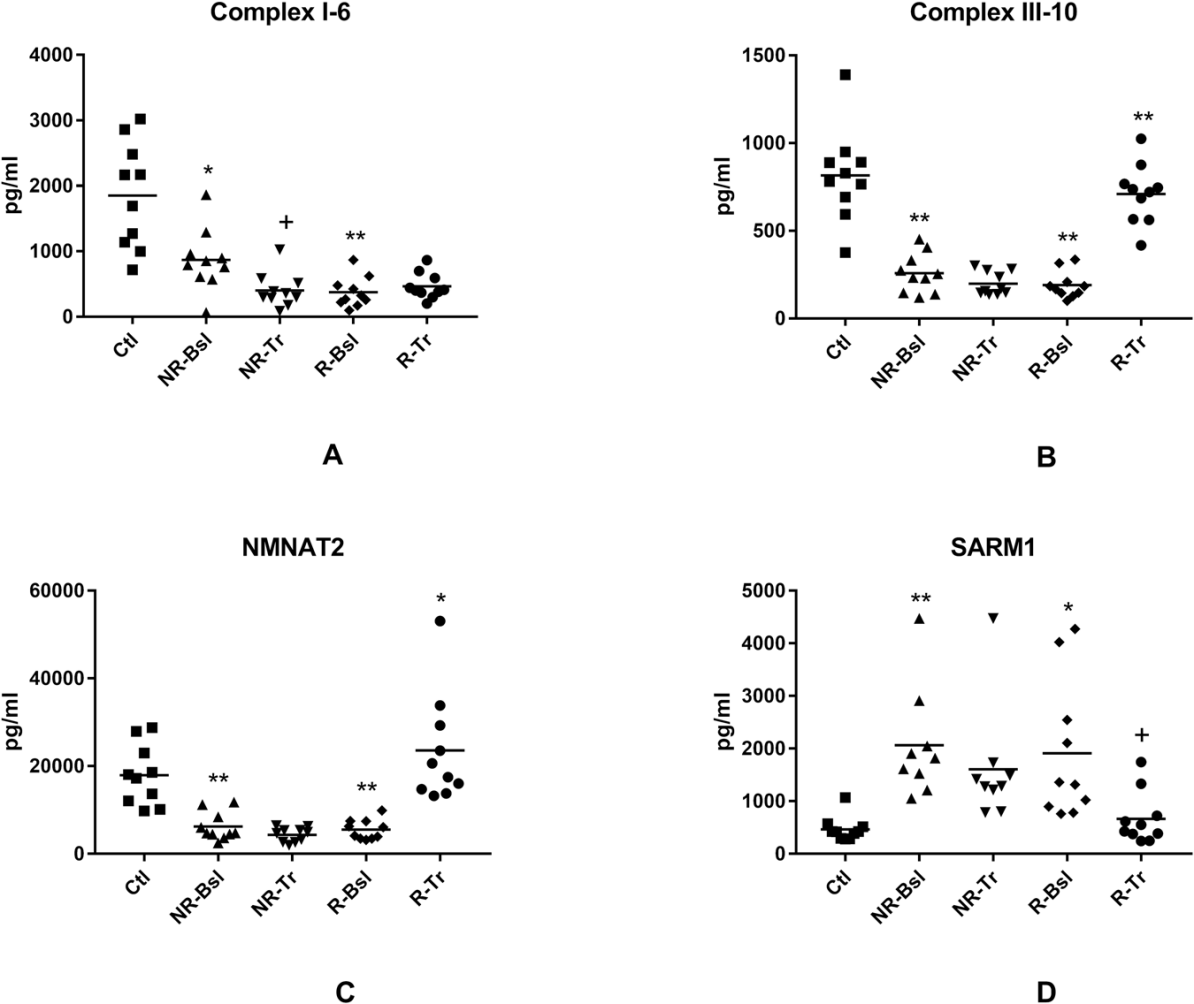
NDEV levels of proteins involved in mitochondrial generation of energy. Statistical methods and symbols are the same as in Fig. 1. The mean ± S.E.M. of Ctl, NR-Bsl, NR-Tr, R-Bsl and R-Tr groups, respectively, were 1852 ± 255 pg/ml, 867 ± 149 pg/ml (0.0037), 401 ± 83 pg/ml (0.0147), 374 ± 74 pg/ml (<0.0001) and 465 ± 63 pg/ml (NS) for Complex I-subunit 6 (**A**); 815 ± 83 pg/ml, 258 ± 35 pg/ml (≤0.0001), 199 ± 21 pg/ml (NS), 191 ± 25 pg/ml (<0.0001) and 710 ± 54 pg/ml (<0.0001) for Complex III-subunit 10 (**B**); 17,908 ± 2171 pg/ml, 6200 ± 1011 pg/ml (<0.0001), 4345 ± 507 pg/ml (NS), 5534 ± 711 pg/ml (<0.0001) and 23,564 ± 3932 pg/ml (0.0014) for NMNAT2 (**C**); and 466 ± 74 pg/ml, 2063 ± 351 pg/ml (0.0002), 2695 ± 1047 pg/ml (NS), 1908 ± 416 pg/ml (0.0031) and 664 ± 156 pg/ml (0.0368) for SARM1 (**D**). Mean ± S.E.M. of levels of the same mitochondrial proteins in NDEV of our previously reported sets (*n* = 10) of controls and participants with a first episode of psychosis [16], respectively, were 1574 ± 246 pg/ml and 542 ± 42.1 pg/ml (0.0007) for Complex I-subunit 6; 1404 ± 112 pg/ml and 635 ± 53.8 pg/ml (<0.0001) for Complex III-subunit 10; 7293 ± 825 pg/ml and 4804 ± 827 pg/ml (0.0471) for NMNAT2; and 974 ± 130 pg/ml and 1160 ± 75.4 pg/ml (NS) for SARM1.

The second class of neuron mitochondrial proteins are critical for energy generation and include the inner membrane electron-transferring complexes NADH dehydrogenase complex I (subunit 6) and cytochrome b-c1 complex III (subunit 10), nicotinamide mononucleotide adenylyl transferase 2 (NMNAT2), which is the penultimate synthetic enzyme required for de novo production of NADH prior to amidation, and the pro-neurodegenerative factor Sterile Alpha and TIR motif-containing protein 1 (SARM1) that has a prominent NADase activity [27, 28] (Fig. 2). The importance of NMNAT2 and SARM1 for energy generation derives from their roles in establishing the mitochondrial total concentration of NADH plus NAD^+^ that is as critical as the NADH/NAD^+^ ratio [27]. The NR-Bsl and R-Bsl levels of both oxidative phosphorylation complexes I and III were significantly lower than the corresponding Ctl levels (Fig. 2A and B). For group R-Tr, the low complex III protein level was completely corrected by treatment, whereas that of complex I protein was not altered significantly. For group NR-Tr, the complex III protein level was not affected by treatment and the level of complex I protein was further decreased significantly. For the two enzymes involved in determining mitochondrial NADH plus NAD^+^ concentration, levels of NMNAT2 were statistically significantly lower (Fig. 2C) and of SARM1 were statistically significantly higher (Fig. 2D) for both the NR-Bsl and R-Bsl groups relative to that of Ctls. Bsl levels of both proteins were normalized for the R-Tr group with no changes for the NR-Tr group.

**Fig. 3:**
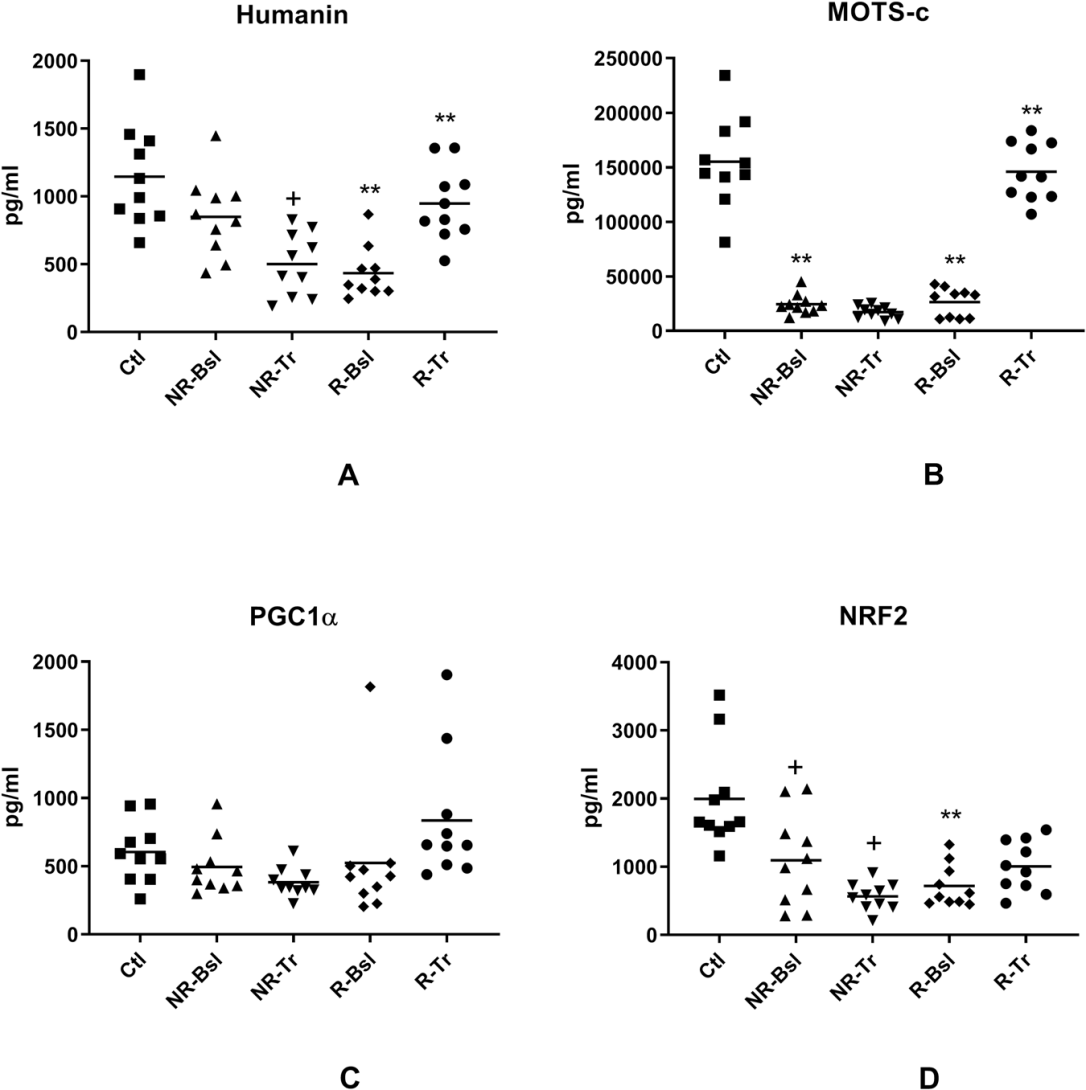
NDEV levels of proteins involved in mitochondrial biogenesis and in mitochondrial regulation of neuronal metabolism and survival. Statistical methods and symbols are the same as in Fig. 1. The mean ± S.E.M. of Ctl, NR-Bsl, NR-Tr, R-Bsl and R-Tr groups, respectively, were 1146 ± 118 pg/ml, 850 ± 93.8 pg/ml (NS), 500 ± 73.4 pg/ml (0.0308), 434 ± 59.9 pg/ml (<0.0001) and 947 ± 86.1 pg/ml (<0.0001) for humanin (**A**); 155,054 ± 13,122 pg/ml, 24,396 ± 2917 pg/ml (<0.0001), 17,126 ± 1757 pg/ml (NS), 26,252 ± 4184 pg/ml (<0.0001) and 145,947 ± 8370 pg/ml (<0.0001) for MOTS-c (**B**); 605 ± 71.2 pg/ml, 494 ± 65.1 pg/ml (NS), 382 ± 33.4 pg/ml (NS), 524 ± 148 pg/ml (NS) and 835 ± 149 pg/ml (NS) for PGC1α (**C**); and 1994 ± 240 pg/ml, 1096 ± 216 pg/ml (0.0122), 563 ± 64.2 pg/ml (0.0439), 719 ± 97.7 pg/ml (0.0001) and 1006 ± 119 pg/ml (NS) for NRF2 (**D**). Mean ± S.E.M. of levels of the same mitochondrial proteins in NDEV of our previously reported sets (*n* = 10) of controls and participants with a first episode of psychosis [16], respectively, were 1752 ± 288 pg/ml and 207 ± 26.1 pg/ml (<0.0001) for humanin; 169,267 ± 12,247 pg/ml and 20,782 ± 2506 pg/ml (0.0007) for MOTS-c; 860 ± 92.5 pg/ml and 675 ± 70.6 pg/ml (NS) for PGC1α; and 1486 ± 223 pg/ml and 556 ±54.6 pg/ml (0.0008) for NRF2.

The third class of neuron mitochondrial proteins include neuroprotective humanin (Fig. 3A) and neuron metabolic regulatory MOTS-c (Fig. 3B) [29–31], which both are encoded by mitochondrial ribosomal RNAs. For the R-Bsl group, the levels of both were statistically significantly lower than those of Ctls and are normalized by treatment for the R-Tr group. For the NR-Bsl group, the level of MOTS-c but not humanin is statistically significantly depressed relative to that of the Ctl group. After treatment, the NR-Tr level of MOTS-c is unchanged and that of humanin is lowered further. The transcription factors PGC-1α and NRF2 are members of a fourth class of neuron mitochondrial proteins that interactively regulate mitochondrial biogenesis through effects on mitochondrial DNA replication [32]. The NDEV levels of PGC-1α showed no differences between any of the clinical groups (Fig. 3C). In contrast, the NDEV levels of NRF2 were statistically lower in the NR-Bsl and R-Bsl groups than in their control groups and neither level was increased in the NR-Tr or R-Tr groups (Fig. 3D).

## Discussion

Immuno-enrichment of plasma extracellular vesicles including exosomes derived from neurons (NDEVs) permitted demonstration of strikingly significant differences between baseline (NR-Bsl and R-Bsl) cargo levels of eleven of the fourteen mitochondrial proteins assessed in MDD as contrasted with those of controls (Ctls) (Figs. 1–3). Only CD81-normalized NDEV levels of TFAM, humanin and PGC-1α showed no differences between NR-Bsl and Ctl groups, whereas only CD81-normalized NDEV levels of TFAM and PGC-1α showed no differences between R- Bsl and Ctl groups (Figs. 1, 3). Statistically significant differences between CD81-normalized NDEV levels of mitochondrial proteins in the NR-Bsl and R-Bsl groups relative to those of Ctls were all decreases except for the increase in SARM-1 (Figs. 1, 2). None of the NDEV levels of mitochondrial proteins in the NR-Bsl group was normalized by treatment, and the decreases in LETM-1, NADH dehydrogenase complex I (subunit 6), humanin and NRF2 levels were instead decreased further. In contrast, all altered NDEV levels of mitochondrial proteins in the R-Bsl group were statistically significantly normalized except those of NADH dehydrogenase complex I (subunit 6) (Fig. 2) and NRF2 (Fig. 3). Pearson Correlation Coefficient analyses did not reveal any statistically significant relationships between HDRS levels of severity of depression and the NDEV concentrations of any mitochondrial protein. However, levels of other variables relevant to depression may correlate with those of NDEV mitochondrial proteins.

There are major quantitative differences between alterations in NDEV levels of mitochondrial proteins in MDD and those observed in FP (Table 3 and figure legends) [16, 17]. For most of the mitochondrial proteins, baseline level decreases relative to controls in the absence of treatment are only modestly quantitatively different between the two diseases, whereas for TFAM and PGC-1α there are no differences in either condition. The greatest differences between mitochondrial proteins in the two disease states are for SARM1 and the tethering proteins. SARM1 is increased four-fold from controls in MDD but unchanged in FP. The tethering proteins present the most complex alterations (Table 3). Levels of both SNPH and MY06 are decreased at baseline in MDD, whereas levels of SNPH are increased two-fold and those of MY06 are unchanged in FP. These tethering proteins are detached as mitochondria are damaged or depleted of critical constituents, move to neuronal cell bodies and enter the endosomal pathway for loading into exosomal NDEVs. In acute neuronal diseases, such as trauma and ischemia, NDEV levels of such proteins may rise or decrease rapidly as a reflection of changes in the removal of damaged proteins. As both MDD baseline and FP single sample plasmas here were obtained at a steady-state later than the immediate onset of disease or treatment, acute transient perturbations are less likely to have contributed to differences which thus should more accurately reflect intraneuronal concentrations. Applying this assumption tentatively for the mitochondria anchored to axonal microtubules by SNPH leads to an initial conclusion that axonal mitochondria are cycling faster than normal in FP and slower than normal in MDD (Table 3). Similarly, these data suggest that presynaptic mitochondria anchored to microfilaments by MY06 also are cycling more slowly in MDD but normally in FP. It is assumed that the differences observed in levels of NMNAT2 and SARM1 would lead to lower mitochondrial concentrations of NADH plus NAD^+^, but this remains to be proven by direct measurements for unextracted NDEVs and intact mitochondria.

NDEV mitochondrial protein findings of this study suggest overall that MDD, as well as FP, is associated with many abnormalities in neuronal mitochondria, including their biogenesis, structure, metabolism, energy generation and production of peptides that protect and regulate normal physiological processes of neurons. The broad normalization of many alterations in NDEV levels of mitochondrial proteins by successful treatment of MDD and the failure of unsuccessful treatment to modify these alterations cannot at this stage unequivocally identify specific underlying mechanisms or drug targets. Nonetheless, some of the alterations in NDEV proteins may be tentatively useful indices of changes in the natural history of disease severity or responses to treatment and may herald early-onset or return of disease before it is clinically apparent. It is also difficult to derive meaningful correlations between the severity of MDD or FP, assessed by psychiatric testing, and the degree of abnormalities of one or more mitochondrial proteins in NDEVs based on initial results from a small number of patients. Findings of larger controlled studies may provide such correlations and identify specifically useful biomarkers and potential novel drug targets.

## References

[1] Ferrari AJ, Somerville AJ, Baxter AJ, Norman R, Patten SB, Vos T (2013). Global variation in the prevalence and incidence of major depressive disorder: a systematic review of the epidemiological literature. Psychol Med.

[2] Ferrari AJ, Charlson FJ, Norman RE, Patten SB, Freedman G, Murray CJ (2013). Burden of depressive disorders by country, sex, age, and year: findings from the global burden of disease study 2010. PLoS Med.

[3] Gaynes BN, Warden D, Trivedi MH, Wisniewski SR, Fava M, Rush AJ (2009). What did STAR*D teach us? Results from a large-scale, practical, clinical trial for patients with depression. Psychiatr Serv.

[4] Lieblich SM, Castle DJ, Pantelis C, Hopwood M, Young AH, Everall IP (2015). High heterogeneity and low reliability in the diagnosis of major depression will impair the development of new drugs. BJPsych Open.

[5] Schatzberg AF (2021). Can target engagement studies miss their targets and mislead drug development?. Am J Psychiatry.

[6] Schmidt HD, Shelton RC, Duman RS (2011). Functional biomarkers of depression: diagnosis, treatment, and pathophysiology. Neuropsychopharmacol: Off Publ Am Coll Neuropsychopharmacol.

[7] Sigitova E, Fisar Z, Hroudova J, Cikankova T, Raboch J (2017). Biological hypotheses and biomarkers of bipolar disorder. Psychiatry Clin Neurosci.

[8] Strawbridge R, Young AH, Cleare AJ (2017). Biomarkers for depression: recent insights, current challenges and future prospects. Neuropsychiatr Dis Treat.

[9] Kennis M, Gerritsen L, van Dalen M, Williams A, Cuijpers P, Bockting C (2020). Prospective biomarkers of major depressive disorder: a systematic review and meta-analysis. Mol Psychiatry.

[10] Sakurai M, Yamamoto Y, Kanayama N, Hasegawa M, Mouri A, Takemura M (2020). Serum Metabolic Profiles of the Tryptophan-Kynurenine Pathway in the high risk subjects of major depressive disorder. Sci Rep.

[11] Le-Niculescu H, Roseberry K, Gill SS, Levey DF, Phalen PL, Mullen J, et al. Precision medicine for mood disorders: objective assessment, risk prediction, pharmacogenomics, and repurposed drugs. Mol Psychiatry. 2021. 10.1038/s41380-021-01061-w. 10.1038/s41380-021-01061-w PMC8505261 33828235

[12] Clay HB, Sillivan S, Konradi C (2011). Mitochondrial dysfunction and pathology in bipolar disorder and schizophrenia. Int J Dev Neurosci.

[13] Picard M, Prather AA, Puterman E, Cuillerier A, Coccia M, Aschbacher K (2018). A mitochondrial health index sensitive to mood and caregiving stress. Biol Psychiatry.

[14] Mustapic M, Eitan E, Werner JK, Berkowitz ST, Lazaropoulos MP, Tran J (2017). Plasma extracellular vesicles enriched for neuronal origin: a potential window into brain pathologic processes. Front Neurosci.

[15] Goetzl EJ (2020). Advancing medicine for Alzheimer’s disease: a plasma neural exosome platform. Faseb J.

[16] Goetzl EJ, Srihari VH, Guloksuz S, Ferrara M, Tek C, Heninger GR (2020). Decreased mitochondrial electron transport proteins and increased complement mediators in plasma neural-derived exosomes of early psychosis. Transl Psychiatry.

[17] Goetzl EJ, Srihari VH, Guloksuz S, Ferrara M, Tek C, Heninger GR (2021). Neural cell-derived plasma exosome protein abnormalities implicate mitochondrial impairment in first episodes of psychosis. Faseb J.

[18] Lindqvist D, Dhabhar FS, James SJ, Hough CM, Jain FA, Bersani FS (2017). Oxidative stress, inflammation and treatment response in major depression. Psychoneuroendocrinology.

[19] Hough CM, Lindqvist D, Epel ES, Denis MS, Reus VI, Bersani FS (2017). Higher serum DHEA concentrations before and after SSRI treatment are associated with remission of major depression. Psychoneuroendocrinology.

[20] Goetzl EJ, Mustapic M, Kapogiannis D, Eitan E, Lobach IV, Goetzl L (2016). Cargo proteins of plasma astrocyte-derived exosomes in Alzheimer’s disease. Faseb J.

[21] Goetzl EJ, Schwartz JB, Abner EL, Jicha GA, Kapogiannis D (2018). High complement levels in astrocyte-derived exosomes of Alzheimer disease. Ann Neurol.

[22] Kang I, Chu CT, Kaufman BA (2018). The mitochondrial transcription factor TFAM in neurodegeneration: emerging evidence and mechanisms. FEBS Lett.

[23] Amanakis G, Murphy E, Cyclophilin D (2020). An integrator of mitochondrial function. Front Physiol.

[24] Filadi R, Pendin D, Pizzo P (2018). Mitofusin 2: from functions to disease. Cell Death Dis.

[25] Kneussel M, Wagner W (2013). Myosin motors at neuronal synapses: drivers of membrane transport and actin dynamics. Nat Rev Neurosci.

[26] Lin MY, Cheng XT, Tammineni P, Xie Y, Zhou B, Cai Q (2017). Releasing syntaphilin removes stressed mitochondria from axons independent of mitophagy under pathophysiological conditions. Neuron.

[27] Stein LR, Imai S (2012). The dynamic regulation of NAD metabolism in mitochondria. Trends Endocrinol Metab.

[28] Jiang Y, Liu T, Lee CH, Chang Q, Yang J, Zhang Z (2020). The NAD(+)-mediated self-inhibition mechanism of pro-neurodegenerative SARM1. Nature.

[29] Guo B, Zhai D, Cabezas E, Welsh K, Nouraini S, Satterthwait AC (2003). Humanin peptide suppresses apoptosis by interfering with Bax activation. Nature.

[30] Lee C, Zeng J, Drew BG, Sallam T, Martin-Montalvo A, Wan J (2015). The mitochondrial-derived peptide MOTS-c promotes metabolic homeostasis and reduces obesity and insulin resistance. Cell Metab.

[31] Cobb LJ, Lee C, Xiao J, Yen K, Wong RG, Nakamura HK (2016). Naturally occurring mitochondrial-derived peptides are age-dependent regulators of apoptosis, insulin sensitivity, and inflammatory markers. Aging (Albany NY).

[32] Gureev AP, Shaforostova EA, Popov VN (2019). Regulation of mitochondrial biogenesis as a way for active longevity: interaction between the nrf2 and pgc-1alpha signaling pathways. Front Genet.

